# Topically Applied Ceramides Interact with the Stratum Corneum Lipid Matrix in Compromised *Ex Vivo* Skin

**DOI:** 10.1007/s11095-017-2288-y

**Published:** 2018-02-06

**Authors:** Tineke Berkers, Dani Visscher, Gert S. Gooris, Joke A. Bouwstra

**Affiliations:** Department of Drug Delivery Technology, Cluster BioTherapeutics, Leiden Academic Centre for Drug Research, Leiden University, Einsteinweg 55, Leiden, 2333 CC The Netherlands

**Keywords:** Ceramides, lipid organization, skin barrier repair, topical formulation

## Abstract

**Purpose:**

To determine whether formulations containing ceramides (including a ceramide with a long hydroxyl acyl chain linked to a linoleate, CER EOS) and fatty acids are able to repair the skin barrier by normalizing the lipid organization in stratum corneum (SC).

**Methods:**

The formulations were applied on a skin barrier repair model consisting of *ex vivo* human skin from which SC was removed by stripping. The effect of formulations on the lipid organization and conformational ordering in the regenerated SC were analyzed using Fourier transform infrared spectroscopy and small angle X-ray diffraction**.**

**Results:**

Application of the formulation containing only one ceramide on regenerating SC resulted in a higher fraction of lipids adopting an orthorhombic organization. A similar fraction of lipids forming an orthorhombic organization was observed after application of a formulation containing two ceramides and a fatty acid on regenerating SC. No effects on the lamellar lipid organization were observed.

**Conclusions:**

Application of a formulation containing either a single ceramide or two ceramides and a fatty acid on regenerating SC, resulted in a denser lateral lipid packing of the SC lipids in compromised skin. The strongest effect was observed after application of a formulation containing a single ceramide.

**Electronic supplementary material:**

The online version of this article (10.1007/s11095-017-2288-y) contains supplementary material, which is available to authorized users.

## Introduction

**Fig. 1:**
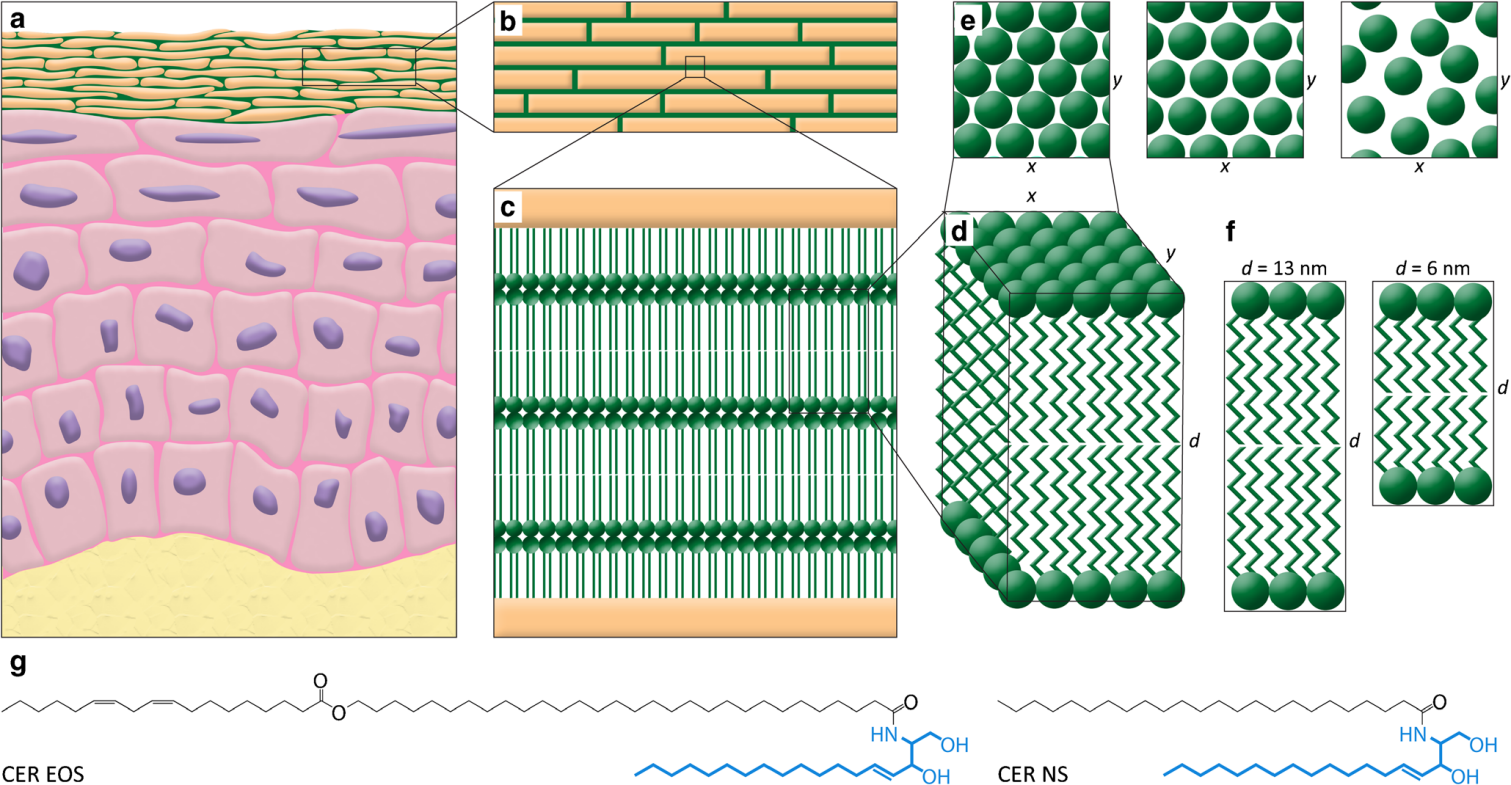
Lipid organization in the SC. **(a)** Schematic overview of the skin morphology, **(b)** The corneocytes are embedded in the lipid matrix in a brick and mortar structure, **(c)** The lipids in the matrix are stacked in lamellae in between the corneocytes, (**d**) Detail of the lipid lamellae, **(e)** Perpendicular to the lamellae, the lipids are organized in a lateral packing. This can be either orthorhombic, hexagonal, or liquid (from left to right), **(f)** The lipid lamellae are stacked on top of each other with a repeat distance (*d*) of either 13 nm (LPP) or 6 nm (SPP), **(g)** Molecular structure and nomenclature of the two CER subclasses used in these studies. The sphingoid base is depicted in blue, and the acyl chain in black.

One of the major roles of the skin is its protection against penetration of irritants, pathogens, and allergens, and prevention of excessive transepidermal water loss (TEWL) avoiding desiccation of the body. The skin barrier function is located in the uppermost epidermal layer, namely the stratum corneum (SC). SC consists of terminally differentiated corneocytes embedded in a lipid matrix. The main lipid classes present in the matrix are ceramides (CERs), fatty acids (FAs), and cholesterol (CHOL). This lipid matrix is important for a proper skin barrier function (1). The intercellular lipids are assembled in two lamellar phases, with a repeat distance of either 13 nm or 6 nm, referred to as the long periodicity phase (LPP) and the short periodicity phase (SPP), respectively (2–6). Within these lamellar sheets, in healthy skin the lipids are mainly packed in an orthorhombic packing, while a small fraction of lipids adopts a hexagonal packing (Fig. 1) (7–10). In several inflammatory skin diseases, a higher fraction of the intercellular lipids assembles in a hexagonal packing, such as in atopic dermatitis (AD) (11). This less dense lipid organization is associated with a reduction in lipid chain length in SC of AD patients and a reduced skin barrier function (11–14). As the lipids play a role in the reduced skin barrier, normalization of the lipid composition and organization may improve the skin barrier function. Normalization of the lipid organization may be achieved by topical application of lipid formulations.

Previously, topical formulations based on vernix caseosa (VC) were developed. VC is a white cream that is developed on the fetal skin during the last trimester of pregnancy. It serves as a lubricant during delivery and it protects the newborn’s skin from dehydration (15). VC contains barrier lipids CERs, FAs, and CHOL, but also other lipids such as super sterol esters, wax esters, squalene, and triglycerides. VC enhances skin barrier repair in mice (16,17).

In order to select the most effective formulation for skin barrier repair, studies are required in which the compositions of the formulations need to be altered in a systematic way. When performing these studies *in vivo*, multiple clinical studies and/or animal studies will be needed. This is very challenging as in clinical studies only a limited number of formulations can be investigated. Furthermore, animal skin is very different from human skin (18). Therefore, we developed an *ex vivo* skin barrier repair (SkinBaR) model (19). In this model, the SC is removed by stripping and subsequently regenerated during an 8-day culturing period. The regenerated SC of the SkinBaR model shows several features that are also observed in SC of AD patients, e.g. a higher fraction of lipids adopting a hexagonal lateral lipid organization and a CER profile that mimics the altered CER subclass composition in AD skin in several aspects (19). Using this model, the influence of topical formulations on the lipid organization and composition of the SC can be examined in *in vitro* settings.

In this study, we used the SkinBaR model to examine whether two CERs of a VC based formulation remain on top of the SC, or are incorporated in the lipid matrix of the SC during repair, thereby improving the lipid organization. We used protiated and partially deuterated CERs. One CER subclass had a very long esterified ω-hydroxy acyl chain (Fig. 1), referred to as CER EOS. The VC based formulation contains a basic formulation (Form^Basic^) and either a single CER subclass or both CER subclasses in combination with a FA. After regeneration of SC, the organization of the lipids in the matrix was examined. Our results demonstrate that the applied formulations containing a single ceramide are able to enhance the formation of an orthorhombic lateral packing and are, at least partly, incorporated in the SC lipid matrix.

## Material and Methods

### Composition and Preparation of Formulations

Two subclasses of synthetic CER were used in these studies (Fig. 1). One CER subclass was the esterified ω-hydroxy acyl chain with 30 carbon atoms in the acyl chain linked to a sphingosine base (18 carbon atoms), referred to as CER EOS according to the classification of Motta *et al.* (20). The other CER was composed of a non-hydroxy acyl chain of 24 carbon atoms linked to a sphingosine base (18 carbon atoms), referred to as CER NS. Deuterated CER NS (dCER NS) consists of a perdeuterated acyl chain and a protiated base.

The basic formulation (Form^Basic^) contained a mixture of super sterol esters, triglycerides (Miglyol 812), squalene, and CHOL in a weight ratio of 13.6:10.1:1.8:1. This basic mixture is supplemented with either CER EOS, CER NS, or dCER NS. For the exact compositions of the formulations and the used abbreviations throughout this paper, see Table I. The use of dCER NS offers the opportunity to selectively detect the deuterated acyl chain of CER NS as CD_2_ vibrations in the infrared spectrum are shifted to lower wavenumbers compared to their protiated counterparts (also see below). In addition to the single CER component in the formulation, a formulation with both CER EOS and CER NS as well as a FA with a chain length of 22 carbon atoms (behenic acid; referred to as FA22) was prepared. Three variations of this formulation were made: i) all lipids were protiated, ii) dCER NS was used instead of CER NS, iii) dFA22 (perdeuterated chain) was used instead of FA22 (see Table I). Finally, the basic formulation (Form^Basic^) without CER or FA was examined.

**Table I:** The Names and Composition of the Various Formulations Used in this Study. Numbers Represent the Weight Percentage of the Components

Name	Basic mixture	CER EOS	CER NS	dCER NS	FA22	dFA22
Form^Basic^	100					
Form^EOS^	98	2				
Form^NS^	98		2			
Form^dNS^	95			5		
Form^COMBI^	94.5	2	2		1.5	
Form^COMBI(dNS)^	94.5	2		2	1.5	
Form^COMBI(dFA)^	94.5	2	2			1.5

The formulations were prepared by dissolving the lipids in either chloroform:methanol (2:1, used for CHOL, CERs, and FA22), chloroform (super sterol esters), or acetone (squalene). Triglycerides were used undissolved. In total 100 mg of the dissolved lipids and triglycerides were mixed in the required ratio in a small glass vial and left to dry under a stream of nitrogen at 40°C for approximately 3 h. In order to distribute the lipids homogeneously in the formulation, the dried lipid mixture was transferred to a mixing tube modified for small scale purposes and mixed for 5 min at 500 rpm using a modified automatic ointment-mixer TopiTec® (WEPA, Germany) (17,21).

### Preparation and Culturing of *Ex Vivo* Skin

Human skin was obtained after surgery from a local hospital according to principles of the Declaration of Helsinki, and used within 12 h after surgery. Stripping and culturing procedures are described in the supplementary material. Formulations were applied in a single dose of about 5 mg/cm^2^ in a metal ring placed on top of the skin at day 0 of the culturing period by rotating movements with the back of a small metal vial (ø = 15 mm) which was covered with one of the formulations (see Table I).

Subsequently, the skin was cultured for 8 days in an incubator during which the SC regenerated. The incubator conditions were 37°C, 90% relative humidity, and 7.2% CO_2_. The culture medium was refreshed twice a week. The composition of the medium has been described in Danso *et al.* (19).

Studies were performed at least in triplicate using the following conditions:

i) Stripped and cultured skin
ii) Stripped and cultured skin + Form^Basic^
iii) Stripped and cultured skin + Form^EOS^
iv) Stripped and cultured skin + Form^NS^ or Form^dNS^
v) Stripped and cultured skin + Form^COMBI^, Form^COMBI(dNS)^ or Form^COMBI(dFA)^

Human skin from the same donor served as control.

After culturing, the skin was harvested and either embedded in paraffin, or SC was isolated (see supplementary material). Isolated SC samples were used to examine the lipid organization or conformational ordering using either Fourier transform infrared spectroscopy (FTIR) or small angle X-ray diffraction (SAXD). Both methods are described in the supplementary material. FTIR provides information about the lateral packing and conformational ordering, while SAXD is used to obtain information about the lamellar organization.

### Statistical Analysis

T-test and one-way ANOVA with a multiple comparisons post hoc test were used to analyze the data using GraphPad prism 7 software (GraphPad software Inc., San Diego, CA, USA).

## Results

### Formulations Do Not Influence the Morphology of Cultured Skin

**Fig. 2:**
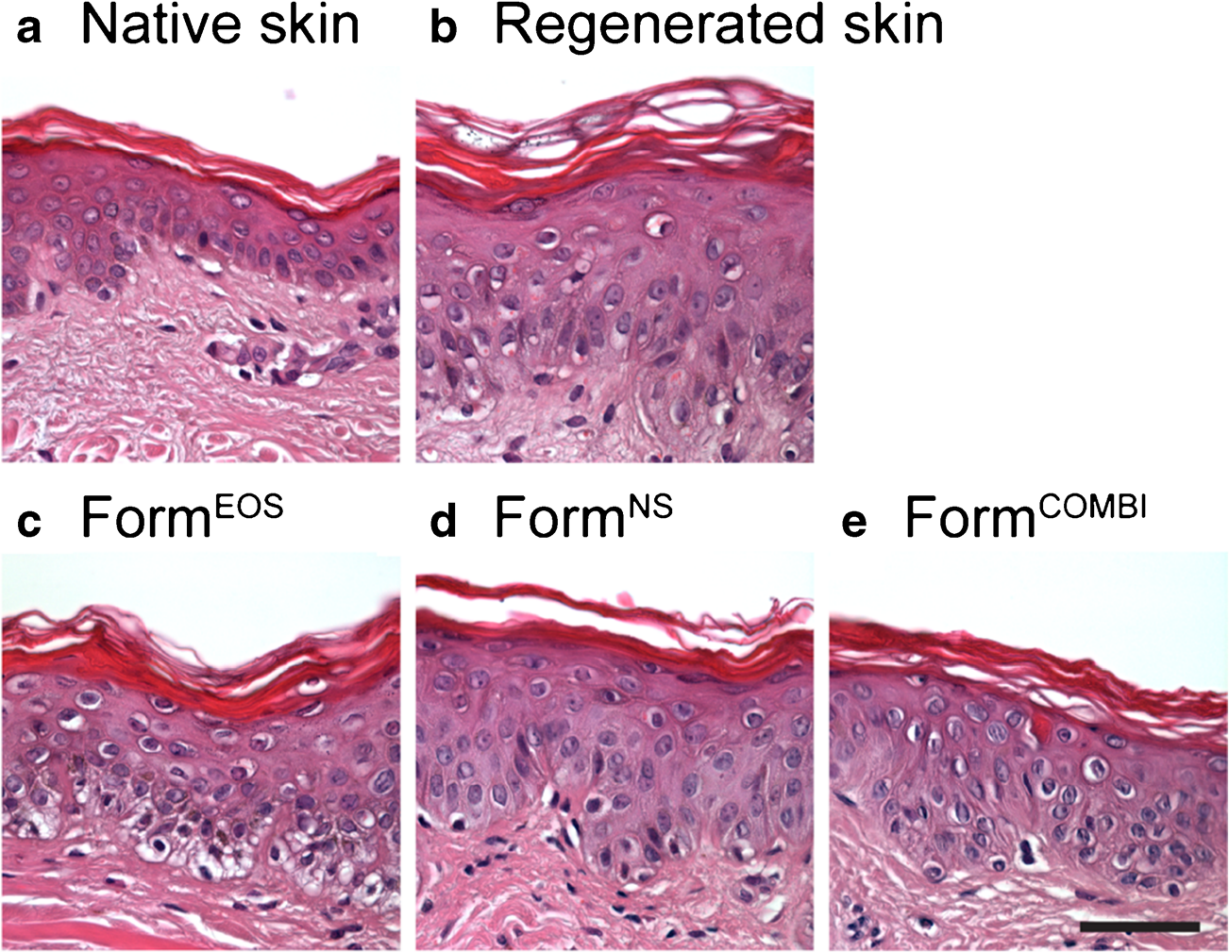
Morphology was examined using HE staining. **(a)** Native human skin, **(b)** Skin with stripped and cultured SC, **(c)** Stripped and cultured skin on which Form^EOS^ was applied, **(d)** Stripped and cultured skin on which Form^NS^ was applied, **(e)** Stripped and cultured skin on which Form^COMBI^ was applied. Scale bar: 50 μm.

As can be observed in Fig. 2, after culturing for 8 days, the epidermis of the stripped and cultured skin was slightly thicker than in native skin and the basal layer was less compact. The morphology of the stripped and cultured skin on which Form^EOS^, Form^NS^, and Form^COMBI^ were applied was comparable to the stripped and cultured skin without formulation. This indicates a viable epidermis after 8 days of culturing, also in the presence of a formulation.

### Ceramides Do Not Influence Lateral Organization of Formulation

CH_2_ rocking vibrations in the FTIR spectra were analyzed in order to examine the lateral lipid organization. When lipids are assembled in a hexagonal packing, only one vibration at around 719 cm^−1^ is visible in the FTIR spectrum. When the lipids adopt a more dense orthorhombic lateral packing, short-range coupling occurs due to interactions of adjacent protiated chains which results in a splitting of the contours. In this case, two peaks are visible at around 719 and 730 cm^−1^. Deuterated chains participating in the same lattice as the protiated lipid chains interfere with the vibrations of the protiated chains, thereby reducing the short-range coupling. This is visible as a reduction in intensity or disappearance of the peak positioned at 730 cm^−1^. In order to be able to compare the intensity of the peaks, all spectra are displayed with a similar ratio between the lowest point at around 715 cm^−1^ and the top at around 719 cm^−1^.

**Fig. 3:**
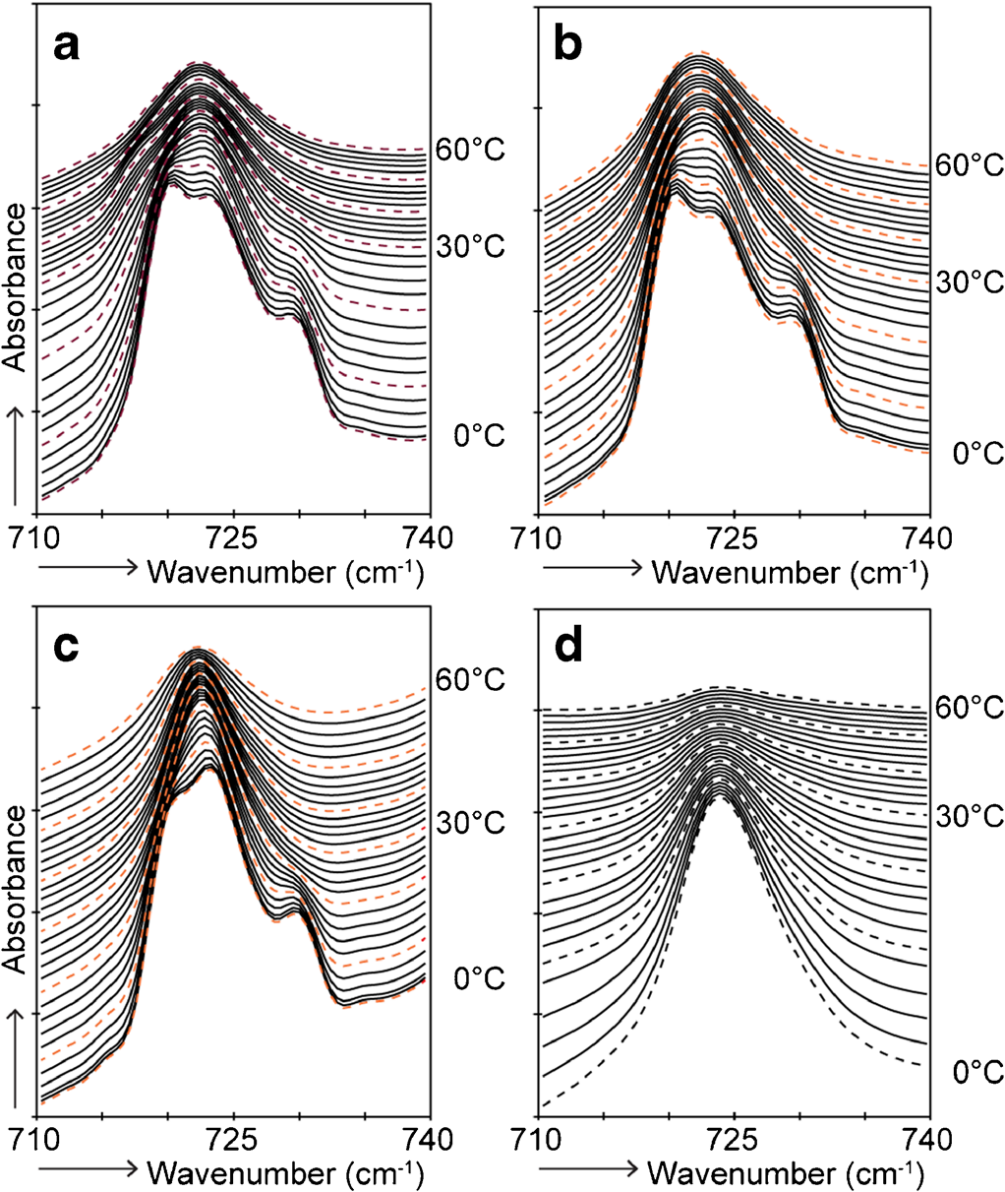
FTIR spectra showing CH_2_ rocking vibrations of lipids in formulations as a function of temperature (0–60°C) **(a)** Form^EOS^, **(b)** Form^NS^, **(c)** Form^dNS^, and **(d)** Triglycerides.

First the spectra of the formulations will be reported. CH_2_ rocking vibrations of Form^EOS^, Form^NS^, and Form^dNS^ showed a strong peak positioned at 719 and a weak peak at 730 cm^−1^, indicating that a small fraction of lipids forms an orthorhombic lateral organization. Furthermore, a third peak was observed at a wavenumber of about 723 cm^−1^ (Fig. 3a-c). This peak can be attributed to the presence of the triglycerides, which showed a single peak of the CH_2_ rocking vibrations at a wavenumber of about 723 cm^−1^ (Fig. 3d). No differences were observed between the various formulations below 10°C. The contour at 730 cm^−1^ in all formulations started to decrease at a temperature of around 10°C and disappeared at 28°C in Form^EOS^ and at 22°C in Form^NS^ and Form^dNS^. This transition is indicative for the disappearance of the orthorhombic lateral packing. Above this temperature all formulation showed similar FTIR profiles. When analyzing Form^Basic^ (no barrier lipids present), the FTIR profile was characterized by a doublet of which the peak at a wavenumber of 730 cm^−1^ disappeared between 10 and 28°C (results not shown).

The profile of pure CER NS was characterized by a single peak located at around 719 cm^−1^, whereas for pure CER EOS a doublet at around 719 and 730 cm^−1^ was observed which was still weakly present at 60°C (Supplementary Figure 1). However, both CER containing formulations and the control formulation showed a similar profile until 10°C, indicating that the CERs do not influence the lateral packing of the formulation at low temperatures as far as it can be detected by FTIR.

### Conformational Ordering of the Lipids is Not Affected by Ceramides in the Formulation

**Fig. 4:**
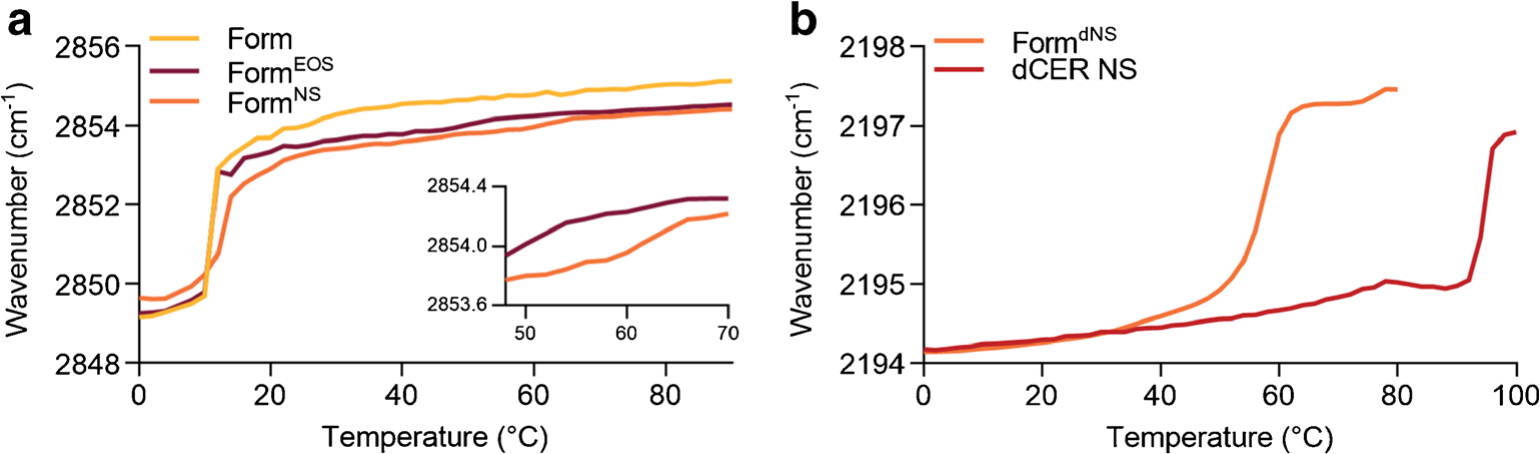
Peak positions of CH_2_ symmetric stretching vibrations and CD_2_ asymmetric stretching vibrations of FTIR spectra plotted as a function of temperature (0–90°C). **(a)** CH_2_ symmetric stretching vibrations of Form^Basic^, Form^EOS^, and Form^NS^. **(b)** Asymmetric CD_2_ stretching vibrations of the deuterated acyl chain of dCER NS and in Form.^dNS^

The conformational ordering of the lipids was analyzed in order to examine the ordered-disordered phase transition of the lipids in the formulations. This was determined by assessing the thermotropic behavior of CH_2_ symmetric and CD_2_ asymmetric stretching vibrations in the FTIR spectrum. When lipids show a high conformational ordering, indicating that the chains are fully extended, CH_2_ and CD_2_ stretching vibration peaks are positioned below a wavenumber of 2850 and 2195 cm^−1^, respectively. Peak positions increase to wavenumbers above 2852 and 2196 cm^−1^, respectively, when the lipids have a high conformational disordering, indicating the presence of a liquid phase. Peak positions of CH_2_ symmetric and CD_2_ asymmetric stretching vibrations of the formulations are plotted against temperature (Fig. 4). The onset transition temperature was determined as described in the supplementary methods section. Figure 4a shows the temperature dependence of Form^Basic^, Form^EOS^, and Form^NS^, which are very similar. At 0°C, the peak positions were located at a wavenumber of around 2849 cm^−1^, indicating an ordered lateral lipid organization. The onset transition temperatures of the ordered-disordered transition of the formulations were approximately 9–10°C. At these temperatures, the CH_2_ symmetric stretching vibrations started to shift to a wavenumber of around 2853 cm^−1^ at 20°C. These increases in wavenumber were steep, representing an ordered-disordered transition. During a further rise in temperature, the CH_2_ stretching vibrations increased gradually until about 2855 cm^−1^ at 90°C. Furthermore, a small shift in wavenumber was observed in the profiles of Form^EOS^ and Form^NS^ at around 50–60°C (see inset). This might be attributed to a change in conformational ordering of CER EOS and CER NS in the formulation (see below).

The temperature dependence of the CD_2_ stretching vibrations of the deuterated acyl chain of pure dCER NS and in Form^dNS^ is depicted in Fig. 4b. At 0°C, the CD_2_ asymmetric stretching peak positions of both Form^dNS^ and pure dCER NS were located at 2194 cm^−1^. The onset transition temperatures were 53°C for Form^dNS^ and 91°C for pure dCER NS. At these temperatures sharp increases in wavenumbers were observed until a wavenumber of around 2197 cm^−1^ was reached at 64°C and 100°C, respectively. The onset transition temperature of pure dCER NS was substantially higher than that of Form^dNS^, indicating that dCER NS interacts with the lipids in the formulation. However, the temperature of transition is not similar to that of the protiated chains indicating that dCER NS is not homogenously mixing with the other protiated components.

### Influence of Topical Formulation on Lateral Lipid Organization of Regenerated SC

**Fig. 5:**
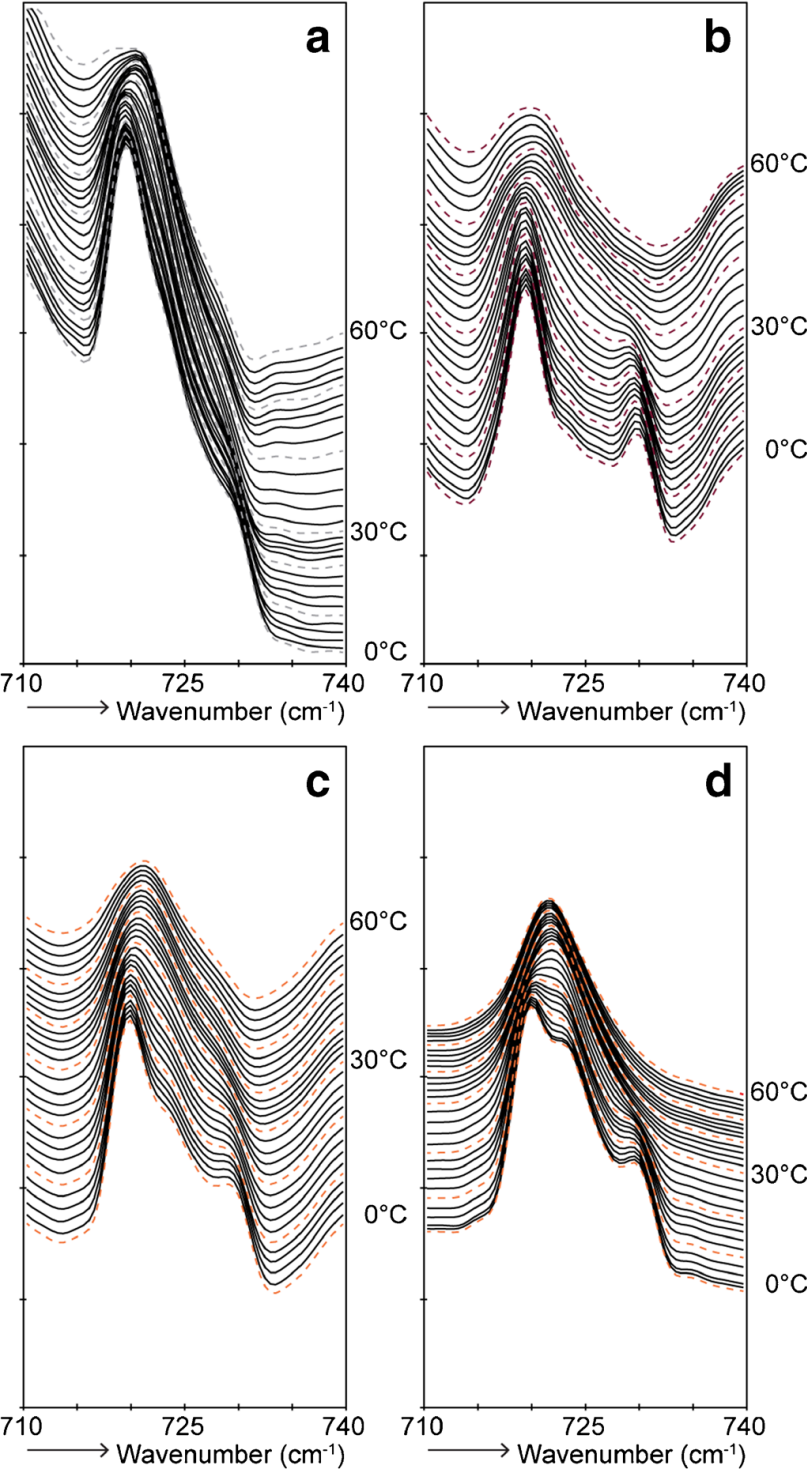
FTIR spectra showing CH_2_ rocking vibrations of SC lipids with and without topical application of formulation as a function of temperature (0–60°C). **(a)** regenerated SC, **(b)** regenerated SC with topical application of Form^EOS^, **(c)** regenerated SC with topical application of Form^NS^, **(d)** regenerated SC with topical application of Form.^dNS^

The CH_2_ rocking vibrations in the FTIR spectrum of native SC showed two strong peaks positioned at 719 and 730 cm^−1^ (Supplementary Fig. 2), indicative for an orthorhombic lateral packing of the lipids. Figure 5a displays CH_2_ rocking vibrations in the FTIR spectra of regenerated SC, which showed a contour with a strong intensity at around 719 cm^−1^ and a peak with a weak intensity at around 730 cm^−1^. This indicates that a higher fraction of lipids adopted a hexagonal lateral packing compared to native SC.

The formulations were applied on the SkinBaR model in order to examine if the CERs from the formulations are incorporated in the SC lipid matrix and furthermore if they participate in the same lattice as SC lipids. After Form^EOS^ was applied during SC regeneration, the peak at 730 cm^−1^ had a stronger intensity (relative to the intensity of the 719 cm^−1^ peak) than that in the spectrum of regenerated SC, indicating a higher level of lipids adopting an orthorhombic lateral packing. After application of Form^NS^ during regeneration of SC, the relative intensity of the peak at 730 cm^−1^ was slightly lower than after application of Form^EOS^, but higher than in the spectrum of the untreated stripped and regenerated SC. A gradual decrease of the peaks around 730 cm^−1^ occurred between 12 and 32°C (regenerated SC), 20°C and 36°C (application of Form^EOS^), and 12°C and 24°C (application of Form^NS^). This indicates that the orthorhombic to hexagonal phase transition took place at lower temperatures than in native SC, but that the presence of CER EOS increased the transition temperature slightly.

The next question is whether CERs from the topically applied formulation form separate domains in the lipid matrix or that these CERs are partitioning in the same lattice as SC lipids. In order to examine this, CH_2_ rocking vibrations were examined after topical application of Form^dNS^ (Fig. 5d). The relative intensity of the peak located at 730 cm^−1^ was similar to the relative intensity of the peak visible after application of its protiated counterpart. This indicates that dCER NS does not partition in the orthorhombic domains. To examine the possibility that CER NS interacts with the hexagonal lateral domains, the temperature dependence of the lateral ordering of the lipids was examined.

### Similar Ordering of Lipids in Regenerated SC in Presence or Absence of a Formulation

**Fig. 6:**
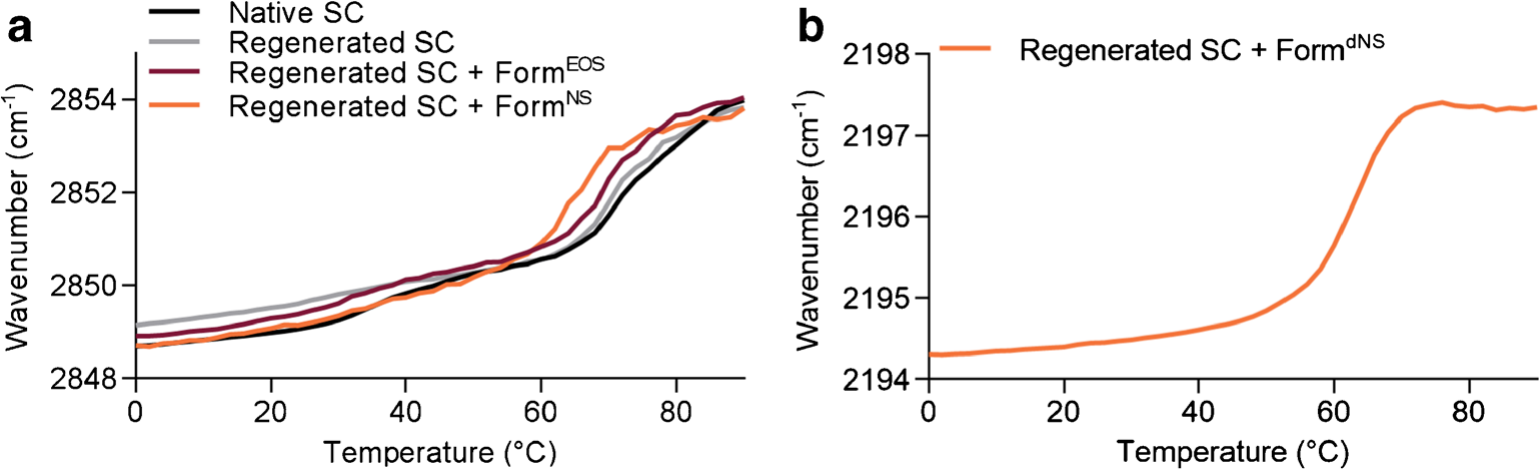
Peak positions of CH_2_ symmetric stretching and CD_2_ asymmetric stretching vibrations of FTIR spectra plotted as a function of temperature (0–90°C). **(a)** Symmetric stretching vibrations of CH_2_ groups in native SC, regenerated SC, and regenerated SC on which Form^EOS^, Form^NS^, or Form^dNS^ was applied **(b)** The CD_2_ asymmetric stretching vibrations of regenerated SC on which Form^dNS^ was applied.

When monitoring the CH_2_ symmetric stretching vibrations of native, regenerated SC, and regenerated SC after application of formulations, very similar temperature dependence was observed (Fig. 6a). A small shift in vibration frequency from 2849 to 2850 cm^−1^ at around 30–40°C was observed in the spectra of native and regenerated SC, which is attributed to the orthorhombic to hexagonal phase transition. The start of the ordered-disordered phase transitions were observed at temperatures of 69.3°C ± 4.0 (*n* = 6) for native SC and 64°C ± 1.5 (*n* = 7) for regenerated SC. The onset transition temperature after application of Form^EOS^ was not affected compared to regenerated SC (62.8°C ± 2.0, *p* = 0.99 (*n* = 3)), whereas after application of Form^NS^ the onset transition temperature was significantly lowered to 58.0°C ± 3.6 (*p* = 0.02 (n = 3)).

Figure 6b depicts the temperature dependence of CD_2_ asymmetric stretching vibrations of regenerated SC on which Form^dNS^ was applied. A gradual increase in wavenumber was observed between 0 and 50°C. The onset transition temperature was 56°C and the end of the transition occurred at 71.0°C ± 1.9. This is a significantly higher temperature than for only Form^dNS^, which was 62.2°C (Fig. 4b, p=0.03). This difference in temperature indicates that there is interaction between dCER NS from the formulation and the SC lipid matrix.

### Combining CER and FA in the Formulation

The final studies focused on a formulation in which both CER EOS and CER NS were included, as well as FA22 (Form^COMBI^). First, the formulation was stored for a period of 6 months at room temperature during which the physical stability was examined at regular time intervals. Polarization microscopy and wide and small angle X-ray diffraction (WAXD and SAXD) were used to examine the physical stability. No crystals were observed in the microscopy images during and after 6 months of storage (results not shown). However, some “Maltese cross” were observed using polarization microscopy, which are indicative for lamellar structures. Additionally, diffraction patterns confirmed the presence of lamellar structures and showed that a fraction of lipids in the formulation adopted an orthorhombic lateral packing at room temperature. No additional peaks were observed, indicating that no crystals were present in the formulation (results not shown).

**Fig. 7:**
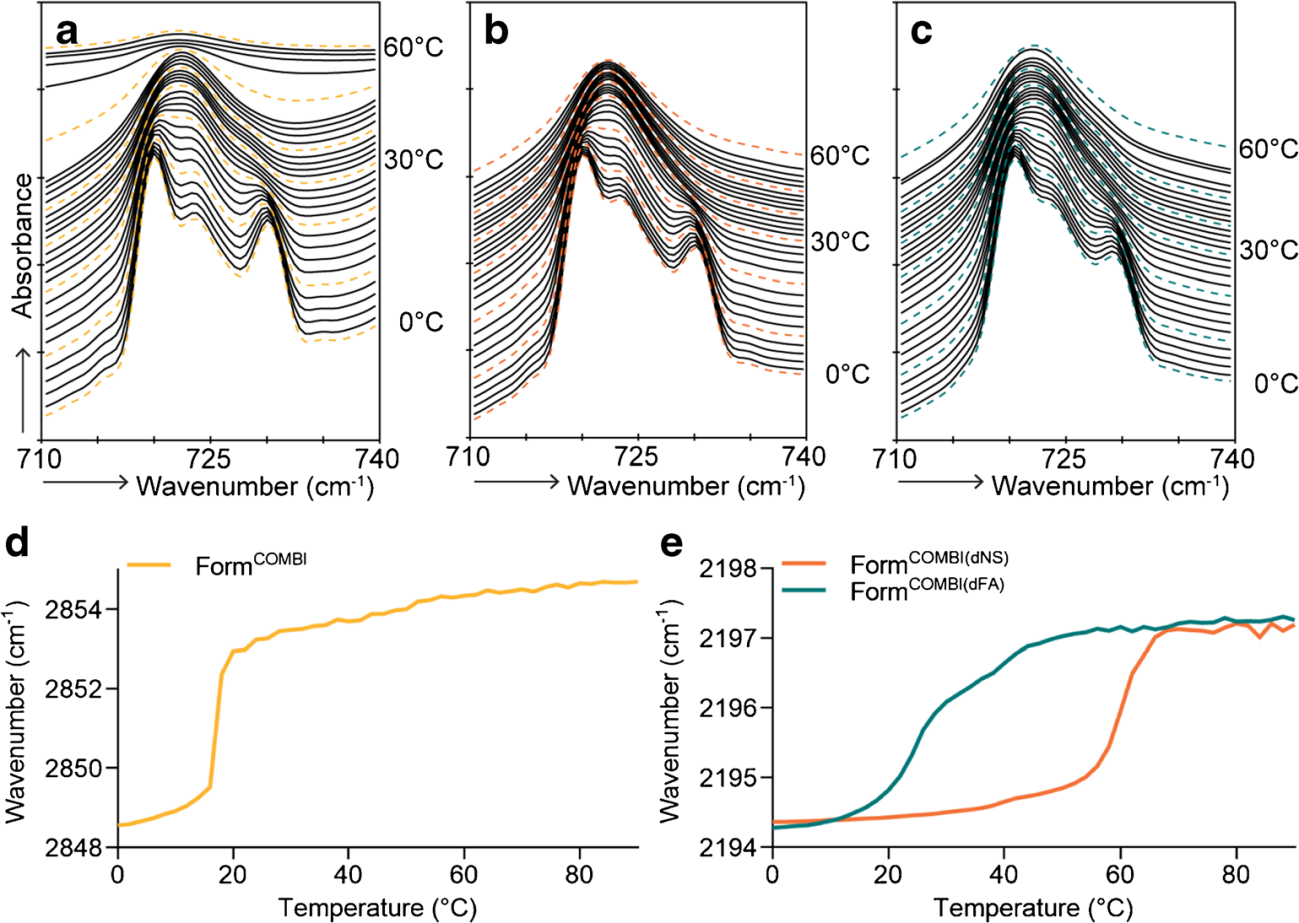
FTIR spectra showing CH_2_ rocking vibrations of lipids in formulations as a function of temperature (0–60°C) and peak positions of CH_2_ symmetric stretching and CD_2_ asymmetric stretching vibrations of FTIR spectra plotted as a function of temperature (0–90°C). **(a)** Form^COMBI^ (only protiated lipids), **(b)** Form^COMBI(dNS)^ (dNS was used) **(c)** Form^COMBI(dFA)^ (dFA22 was used), **(d)** Symmetric stretching vibrations of CH_2_ groups in Form^COMBI^ **(e)** The CD_2_ asymmetric stretching vibrations of Form^COMBI(dNS)^ and Form.^COMBI(dFA)^

The lateral lipid organization of Form^COMBI^ with only protiated lipids was examined using FTIR (Fig. 7a). The contour at 730 cm^−1^ disappeared in the same temperature range as in Form^EOS^ and Form^NS^. However, the relative intensity of the contour at 730 cm^−1^ was higher compared to a formulation with only one CER subclass, indicating that a higher fraction of lipids adopted an orthorhombic lateral packing in Form^COMBI^.

When substituting CER NS by dCER NS (Fig. 7b) or FA22 by dFA22 (Fig. 7c) in the formulation, the relative intensity of the peak at 730 cm^−1^ was reduced compared to the formulation with only protiated lipids. This suggests that a fraction of the deuterated lipids partitions in the orthorhombic lattice formed by the protiated lipids of the formulation (see above).

The thermotropic behavior of the CH_2_ stretching vibration in the spectrum of Form^COMBI^ with only protiated lipids was highly comparable to the formulations with only one CER. However, the onset transition temperature was increased to 16°C (Fig. 7d). When replacing FA22 by dFA22 the onset transition temperature of the CD_2_ asymmetric stretching vibrations was at 18°C and took place over a large temperature range, indicating an order-disorder transition over a large temperature interval. The onset transition temperature was somewhat higher than the formulation with protiated lipids, but lower than the onset transition temperatures of pure dFA22 (22). When substituting CER NS by dCER NS, the onset transition temperature was 55°C (Fig. 7e), which is also substantially higher than in the protiated formulation in which all lipids contribute to this shift, but a lower temperature than for the same transition in pure dCER NS. This indicates that a fraction of dFA and dCER NS interacts with protiated lipids, but the dCER NS and dFA do not show a concerted order-disorder transition with most of the protiated lipids. This may demonstrate different structural domains in the formulation, most probably FA-rich and CER-rich domains.

### Incorporation of Lipids in Regenerated SC

**Fig. 8:**
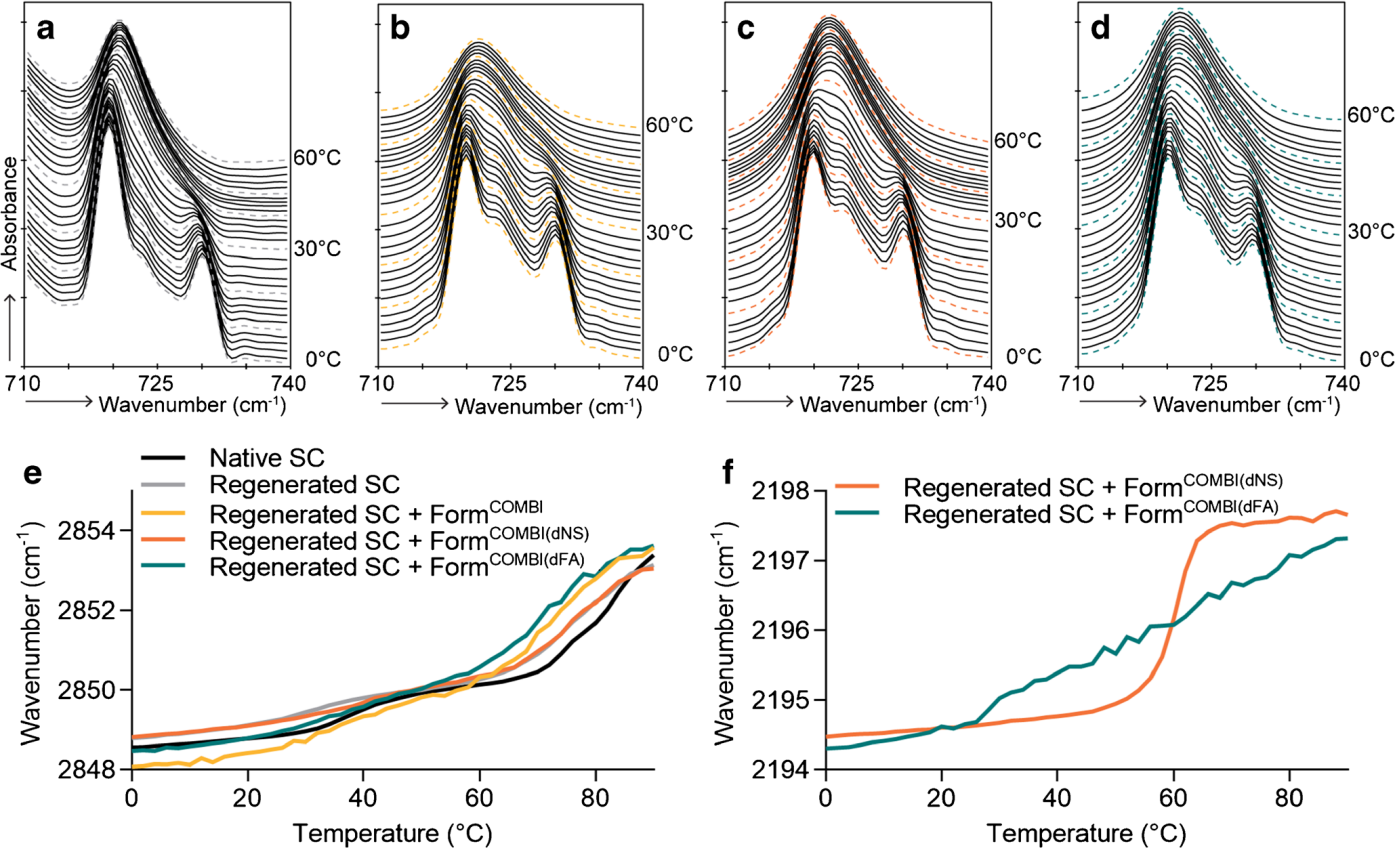
FTIR spectra showing CH_2_ rocking vibrations of SC lipids after application of formulations as a function of temperature (0–60°C) and peak positions of CH_2_ symmetric stretching and CD_2_ asymmetric stretching vibrations of FTIR spectra plotted as a function of temperature (0–90°C). **(a)** Regenerated SC, **(b)** Regenerated SC after application of Form^COMBI^ (only protiated lipids), **(c)** Regenerated SC after application of Form^COMBI(dNS)^, **(d)** Regenerated SC after application of Form^COMBI(dFA)^, **(e)** Symmetric stretching vibrations of CH_2_ groups in regenerated SC after application of Form^COMBI^ **(f)** The CD_2_ asymmetric stretching vibrations of regenerated SC after application of Form^COMBI(dNS)^ and Form.^COMBI(dFA)^

After application of Form^COMBI^ on regenerating SC, the CH_2_ rocking vibrations in the FTIR spectrum showed two strong peaks at around 719 and 730 cm^−1^, comparable to regenerated SC on which Form^EOS^ was applied (Fig. 8). No major differences were observed after application of a formulation containing dCER NS instead of CER NS. However, after the use of dFA22 instead of FA22, the intensity of the peak at 730 cm^−1^, relative to the intensity at 719 cm^−1^, was decreased (Fig. 8d). This indicates that the FA22 from the formulation participates at least partly in the orthorhombic packing with the protiated SC lipids.

The temperature dependence of the CH_2_ symmetric stretching vibrations after application of the formulations on regenerating SC was comparable to the temperature dependence of regenerated SC without formulation. When focusing on Form^COMBI(dNS)^, the onset of transition of the CD_2_ asymmetric stretching vibrations and CH_2_ symmetric stretching vibration after application on SC is very similar, but the transition occurs over a smaller temperature range in the CD_2_ asymmetric stretching vibrations (compare Fig. 8e and f). This temperature range is similar to that of the CD_2_ stretching vibrations in the formulation. However, the thermotropic behavior of the CD_2_ asymmetric stretching vibrations of Form^COMBI(dFA)^ shows a completely different profile. The ordered-disordered transition starts at around 26°C, as opposed to 16°C for the formulation only, and the transition occurs in a very broad temperature range. This indicates that at least a fraction of the dFA from the formulation interacts with the SC lipids, which show an ordered-disordered phase transition at around 60–70°C. Furthermore, the contours of the CD_2_ symmetric stretching vibrations indicate the presence of two vibration modes (see Supplementary Fig. 3) with a main peak at a wavenumber of around 2090 cm^−1^ and a shoulder around 2086 cm^−1^.

### Application of CER Containing Formulation Does Not Influence Lamellar Lipid Organization

The lamellar organization in the lipid matrix was studied using SAXD. The peak positions are indicative for the spacing of the lamellae in the SC. The diffraction patterns of native and regenerated SC with or without formulation are shown in Supplementary Fig. 4. All SC samples showed a main peak at a q-position of about 1.0 nm^−1^, which is attributed to the 1st order SPP peak and the 2nd order LPP peak. In some samples, a small difference in shape of the peak was observed, but the peak position was not changed. Furthermore, sometimes a peak at q = 1.9 nm^−1^ was observed, which corresponds to phase separated crystalline CHOL that is present in SC.

## Discussion

In the present study, the SkinBaR model that mimics several aspects of the lipid organization in AD skin was used to investigate the effect of topical skin barrier repair formulations. We demonstrate that after application of formulations containing barrier lipids and one CER subclass, a higher fraction of SC lipids adopts an orthorhombic lateral packing in the regenerated SC, mimicking more closely the lipid organization in native human skin. However, when two CER subclasses were combined with a FA, this effect was not observed.

In inflammatory skin diseases, like AD, the skin barrier function is impaired as indicated by an increased TEWL (23,24). Besides changes in protein levels in the epidermis in these skin diseases, an altered lipid composition and organization compared to healthy skin has also been reported (13,23–31). Particularly the importance of the lipids for the skin barrier has been indicated by a strong correlation between increased TEWL and reduced chain length of both CER and FA, and a reduced fraction of lipids forming an orthorhombic lateral packing (11,13,14). These results show that the skin barrier might be improved by normalizing the lipid composition and organization. Previously, several skin barrier repair mixtures containing skin barrier lipids CER, FA, and CHOL were reported (32–36). However, the precise composition of the formulation is often not described (34–36). Furthermore, most studies focus on TEWL and/or skin hydration as end point measurements (32–34,36). None of these studies investigated the effect of the formulations on the lipid composition and/or organization in SC. This demonstrates that little is known about the key interactions of the barrier lipids applied in the formulations and the lipid matrix in the SC, which may be an important underlying mechanism for skin barrier repair. Therefore, additional research is needed to provide detailed insights in these interactions and select the optimal skin barrier repair formulation.

Previously, it has been reported that both natural and synthetic VC applied on tape-stripped mouse skin enhanced skin barrier repair *in vivo* (16,17). Based on these findings, in the present study we developed formulations containing the barrier lipids CER, FA, and CHOL and examined their effect on the SC lipid organization in an *ex vivo* human skin barrier repair (SkinBaR) model. This model mimics more closely the morphology, the lipid organization, and the lipid composition of AD than that in mice skin: An increased level of short chain CERs and a fraction of unsaturated CERs are present in SC of the SkinBaR model, similarly as in SC of AD skin (19,37).

In the present study, the CER containing formulation was topically applied on regenerating SC of the SkinBaR model in order to examine whether the lipid organization could be normalized toward that in native human SC and whether barrier lipids in the formulation participate in the SC lipid matrix or mainly remain on the skin surface. In order to examine this, FTIR studies were executed using protiated and (if available) a deuterated CER or FA in the formulations. The use of deuterated lipids in the formulations applied on the SkinBaR model facilitates more detailed analysis of the interactions between the deuterated lipids from the formulations and the SC lipid matrix. First, the effect of only one CER subclass in the formulation was examined. Subsequently, a formulation with a single FA in combination with the two CER subclasses was studied.

### Formulations

The physical stability of the formulations was examined during a period of 6 months. During the storage, no crystals were observed. However, when CER NP or CER EOP were introduced in the same formulation, crystals were formed (results not shown). Therefore, all experiments were performed using CER NS and CER EOS.

#### CERs

Examination of the lateral lipid organization and conformational ordering of the lipids in the formulation indicated that the presence of both the orthorhombic domains and the ordered phase start to disappear at around 10°C. To examine in detail the interaction between CERs and the formulation components, deuterated CER NS was used. Several observations suggest that dCER NS interacts with the other components in the formulation, namely i) the CD_2_ stretching frequencies show that the onset transition temperature to a fluid phase is lower for dCER NS in Form^dNS^ than that of pure dCER NS, and ii) no crystals are observed in the formulation during a period of 6 months. However, replacing CER NS by dCER NS did not result in a decrease in the CH_2_ rocking vibration contour at 730 cm^−1^. This suggests that dCER NS does not participate in the orthorhombic lattice in the formulation. As CER NS itself forms a hexagonal lateral packing, it may be that CER NS is located in domains with a hexagonal packing. Supplementation of CER EOS did increase the fraction of lipids forming an orthorhombic packing and increases the stability of the orthorhombic packing slightly. As no deuterated CER EOS is available, it could not be determined whether CER EOS is intercalated in the orthorhombic matrix.

#### FAs and CERs

In addition to the CERs, FA is another candidate barrier lipid to incorporate in a formulation. In previous studies, FAs with varying chain length were incorporated in the formulation (21). FA22 showed the most abundant change toward a more orthorhombic lateral packing and was therefore selected for the present studies.

An important question to answer is whether a combination of CER EOS and CER NS with FA22 in the formulation, Form^COMBI^, results in an increased fraction of lipids adopting an orthorhombic packing. The results obtained from CH_2_ rocking vibrations in the FTIR spectra indicated an increased fraction of lipids adopting an orthorhombic lateral packing compared to a formulation with only one CER subclass or FA (21). This is in accordance with previous results showing that both CER EOS and (very) long chain FA are important for the formation of orthorhombic domains (38–41).

To obtain more detailed information about the interaction of CER and FA in the formulation, either CER NS or FA22 was replaced by its deuterated counterpart. Participation of at least a fraction of both dCER NS and dFA22 in the orthorhombic lattice in the formulation was demonstrated by the reduced relative intensity of the rocking vibration peak located at 730 cm^−1^ compared to the formulation with only protiated lipids. Interaction of dFA with the other barrier components in the formulation is further demonstrated by an increased onset transition temperature of the CD_2_ stretching vibrations in Form^COMBI(dFA)^ compared to that of only dFA in the formulation and decreased compared to that of pure dFA (21,22). However, a large difference in onset transition temperature of dCER NS (Form^COMBI(dNS)^) with dFA22 (Form^COMBI(dFA)^) was observed. This strongly suggests that at least two different types of domains of barrier lipids are present in the formulation, most probably both containing CERs as well as FAs, but the domains may be either dCER NS rich (high order-disorder transition temperature) or dFA rich domains (lower order-disorder transition temperature).

### Interactions Between Formulations and Stratum Corneum

After having characterized the formulations, we examined the interactions of the formulations with the regenerating SC. After application of Form^EOS^ or Form^NS^ on regenerating SC, a higher fraction of lipids adopted an orthorhombic lateral packing as indicated by a higher relative intensity of the CH_2_ rocking peak at 730 cm^−1^ in the FTIR spectrum. The increase in intensity was more pronounced with CER EOS than CER NS in the formulation, most probably due to the long acyl chain of the former. The increase in the 730 cm^−1^ relative intensity for Form^NS^ is only encountered when the FTIR spectrum in the untreated regenerated SC does not exhibit a 730 cm^−1^ peak, demonstrating the presence of only a hexagonal lateral packing. Based on the similar relative intensity of the rocking vibrations after application of Form^NS^ and Form^dNS^ there is no evidence that CER NS participates in the orthorhombic packing of the SC lipid matrix. Possibly CER NS participates in the hexagonal domains. Therefore, we also examined the lipid ordering of the SC lipid matrix after application of Form^NS^ and Form^dNS^. After application of both formulations on regenerating SC, a comparable onset transition temperature was observed. This onset transition temperature was also comparable to that of Form^dNS^ alone. However, there is a difference in temperature at which the ordered-disordered transition terminates, namely 62.2°C for Form^dNS^ and 71.0°C for regenerated SC treated with Form^dNS^. This indicates that at least a part of the dCER NS interacts with lipids in the SC matrix.

Finally, Form^COMBI^ was applied on regenerating SC and analyzed in the same manner. Application of Form^COMBI^, in which CER EOS, CER NS, and FA22 are combined, did not increase the fraction of lipids adopting an orthorhombic lateral packing compared to untreated regenerated SC. However, the absence of an increase in the formation of the orthorhombic packing is probably due to the high fraction of lipids forming an orthorhombic packing in the untreated regenerated SC, making it a bigger challenge to increase the fraction of lipids forming an orthorhombic packing. The decreased relative intensity of the peak at 730 cm^−1^ after replacing FA22 by dFA22 in Form^COMBI^ indicates that dFA22 participates in the orthorhombic lattice and is most likely intercalated in the SC matrix. Previously, we have reported the application of a formulation containing only one FA (21). In that paper we show that dFA participate in the orthorhombic lattice and that it interacts with the SC lipid matrix (21). It seems that this effect has been slightly reduced when CERs were added to the formulation. Possibly, CER NS and CER EOS interfere with the interaction between FA and the SC lipid matrix probably by stabilizing the FA in the formulation and reducing the partitioning into the orthorhombic lipid matrix in the SC. In contrast, the reduced peak intensity was not observed when CER NS was replaced by dCER NS, suggesting that CER NS is not intercalated in the orthorhombic domains in the SC lipid matrix when FA22 and CER EOS are also present in the formulation. From the stretching vibrations there is also no evidence that CER NS is present in the SC lipid matrix. However, as the ordered-disordered transition of dCER NS in the formulation is in a similar temperature range as that of the SC lipid matrix, some of the CER NS may still intercalate in the hexagonal packing in the lipid matrix, or CER NS may be present in the skin furrows and may still influence barrier repair.

Several other formulations consisting of CERs, FA, and CHOL report enhanced barrier repair for formulations containing CER subclass NP (42–44). However, mainly TEWL was used as skin barrier repair parameter, and interaction with SC lipid matrix was not examined. In our formulation CER NP crystallizes and therefore CER NP was not suitable to be used in the present study. Other studies report formulations in which CER subclass AdS was used as CER component. Topical application of these formulations resulted in decreased TEWL values and a higher skin hydration after 4 weeks (45,46). Furthermore, addition of CER AdS to the culture medium of reconstructed human skin resulted in increased CER content, mainly caused by an increase of CER EOS, NS, and NP (47).

## Conclusion

In conclusion, we show that CERs interact with the other components of a lipid formulation, resulting in a higher fraction of lipids adopting an orthorhombic lateral packing compared to a formulation without CERs. These interactions were also observed for FA and CERs when both lipid classes were present in the formulation.

After application of the formulation containing either CER EOS or CER NS on regenerating SC of the SkinBaR model, a denser lipid packing was observed, suggesting that CERs from the formulation interact with the SC lipid matrix. When studying a formulation with the three barrier components FA, CER NS and CER EOS, there is strong evidence that FA is interacting with the orthorhombic domains in the SC lipid domains, while there is no clear indication that CER NS is intercalated within the SC lipid matrix.

## Electronic Supplementary Material

ESM 1 (DOCX 3664 kb)

## Abbreviations

AD: Atopic dermatitis
CER: Ceramide
CHOL: Cholesterol
FA: Fatty acid
Form^Basic^: Basic formulation without CERs
Form^COMBI^: Formulation with CER EOS, CER NS, and FA22
Form^EOS^: Formulation with CER EOS
Form^(d)NS^: Formulation with (deuterated) CER NS
FTIR: Fourier transform infrared spectroscopy
LPP: Long periodicity phase
SAXD: Small angle X-ray diffraction
SC: Stratum corneum
SkinBaR: Skin barrier repair
SPP: Short periodicity phase
TEWL: Transepidermal water loss
VC: Vernix caseosa
