# Supplementary material for: Topically Applied Ceramides Interact with the Stratum Corneum Lipid Matrix in Compromised Ex Vivo Skin
Source: Pharm Res. 2018 Feb 6;35(3):48. doi: 10.1007/s11095-017-2288-y (PMC5801391; doi:10.1007/s11095-017-2288-y)
Supplement: Supplementary file 1 — (DOCX 3664 kb) [file 11095_2017_2288_MOESM1_ESM.docx]

**Supplementary material**

**Materials and Methods**

Chemicals

Super sterol esters were kindly provided by Croda (Cowick Hall, UK), triglycerides (Miglyol 812) were supplied by Cremer Oleo (Witten, Germany). CER EOS30 and CER NS24 were provided by Evonik (Essen, Germany). Deuterated CER NS24 was kindly supplied by Evonik Industries AG (Essen, Germany), perdeuterated behenic acid was acquired from Cambridge Isotope Laboratories (Tewksbury, MA, USA). Cyanoacrylate (Bison, Goes, the Netherlands) was bought locally. Xylene and methanol were obtained from Biosolve (Valkenswaard, the Netherlands), 4% buffered formaldehyde was purchased from Added Pharma (Oss, the Netherlands), paraffin, haematoxylin, and eosin were acquired from Klinipath (Duiven, the Netherlands). DMEM, Ham’s F12, and penicillin/streptomycin were purchased from Fisher Scientific (Waltham, Massachusetts, USA). Squalene, cholesterol, bovine serum albumin, sodium bromide, ethanol, acetone, trypsin, trypsin inhibitor, selenious acid, hydrocortisone, isoproterenol, L-carnitine, L-serine, insulin, α-tocopherol acetate, vitamin C, arachidonic acid, linoleic acid, and palmitic acid were bought from Sigma-Aldrich (Zwijndrecht, the Netherlands). Chloroform was obtained from Macron Fine Chemicals (Gliwice, Poland). All solvents were HPLC grade or higher.

Stripping and culturing of *ex vivo* skin

Before dermatoming to a thickness of 600 µm (D80 Dermatome, Humeca, Borne, the Netherlands), the subcutaneous fat was removed, and the skin was wiped with 70% EtOH and Millipore water. SC was removed by sequential stripping as described before (1). Briefly, skin was punched (ø=26 mm) and stretched on a custom made clamping device. SC was removed by preheated (40°C) metal cylinder with preheated cyanoacrylate. During each stripping the metal cylinder was applied on the skin for 2 minutes with a pressure of 0.7 kg/cm^2^. This procedure was repeated until the SC was removed which is indicated by a shiny appearance of the skin.

The detailed culturing procedure has been described previously (1, 2). In short, the stripped skin was placed on top of a cotton pad and a transwell filter insert (Corning Life sciences, Amsterdam, Netherlands) in a deep 6-well culturing plate (Organogenesis, Canton, MA, USA). A metal ring (inner diameter 15 mm) was placed on the SC side of the skin.

Isolation of SC

Trypsin was used to isolate SC from cultured skin. The skin was kept at 4°C overnight in 0.1% trypsin solution in PBS, followed by one hour at 37°C. SC was peeled off and washed in 0.1% trypsin inhibitor solution in PBS. SC sheets were cleaned twice in Millipore water and stored over silica gel under argon in the dark until use. SC was used for either infrared spectroscopy measurements or X-ray diffraction measurements.

Fourier transform infrared spectroscopy

The conformational ordering and the lateral packing of the lipids in the SC matrix and the formulations was examined using Fourier transform infrared spectroscopy (FTIR). SC samples were hydrated over a 27% NaBr solution in D_2_O for 24 hours at room temperature. FTIR spectra were recorded using a Varian 670-IR FTIR spectrometer (Agilent Technologies, Santa Clara, USA), equipped with a broadband mercury-cadmium-telluride detector. A hydrated SC sample or a formulation was placed between two AgBr-windows and measured in transmission mode. Samples were put under a continuous purge of dry air starting 30 minutes before the beginning of the measurement. Spectra were obtained as a co-addition of 128 scans at 1 cm^-1^ resolution during 2 minutes, during which the sample temperature was increased from 0 to 90°C at a heating rate of 0.5°C/min. Resolutions Pro 4.1 (Varian Inc.) software was used to analyze the data (3).

The peak positions of the CH_2_ symmetric and CD_2_ asymmetric stretching vibrations in the FTIR spectra were used to determine the onset temperature of the ordered-disordered transition. Two regression lines were fitted to the linear parts of the graph obtained after plotting the peak positions against temperature. The intercept of the two regression lines determines the onset transition temperature, as described before (2). The end of the ordered-disordered phase transition was determined in the same manner as the onset transition temperature.

Small angle X-ray diffraction

The lamellar lipid organization was examined using small angle X-ray diffraction (SAXD). Measurements were performed at the European Synchrotron Radiation Facility (ESRF, Grenoble, France) at station BM26B. The SC samples were hydrated over a 27% NaBr solution during 24 hours prior to the measurements. SC samples were carefully oriented parallel to the X-ray beam in a custom made sample holder. SAXD patterns were detected with a Pilatus 1M detector at room temperature for a period of 5 or 10 min as described earlier (4). The scattering vector (q) was calculated from the scattering angle (Θ) and the wavelength (λ) by *q=4π sin Θ/λ*. From the position of the peak maxima (q), the spacing of the lamellar phase can be calculated using *2π/q*. A peak positioned at a lower q-value corresponds to a larger spacing of the lipid lamellae.

**Results**


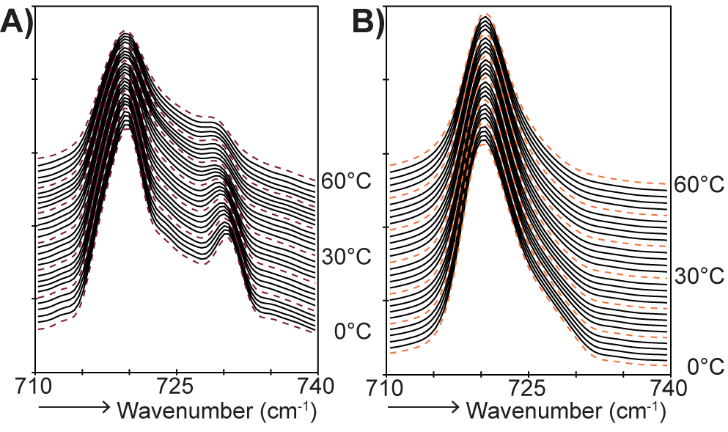


Supplementary Figure 1. FTIR spectra showing CH_2_ rocking vibrations of pure CER as a function of temperature (0-60°C) A) CER EOS, characterized by a doublet with vibrations at around 719 and 730 cm^-1^ B) CER NS, characterized by a singlet at a wavenumber of around 719 cm^-1^.


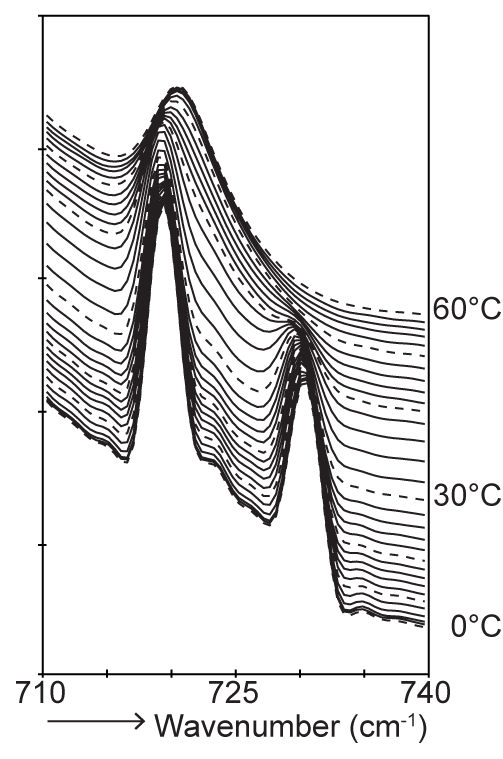


**Supplementary Figure 2.** FTIR spectrum showing CH_2_ rocking vibrations of native SC. Two strong peaks are observed at wavenumbers of around 719 and 730 cm^-1^. The intensity of the peak at 730 cm^-1^ started to decrease at a temperature of around 30°C and the contour disappeared at 48°C. This change in rocking profile is indicative for the orthorhombic to hexagonal phase transition.


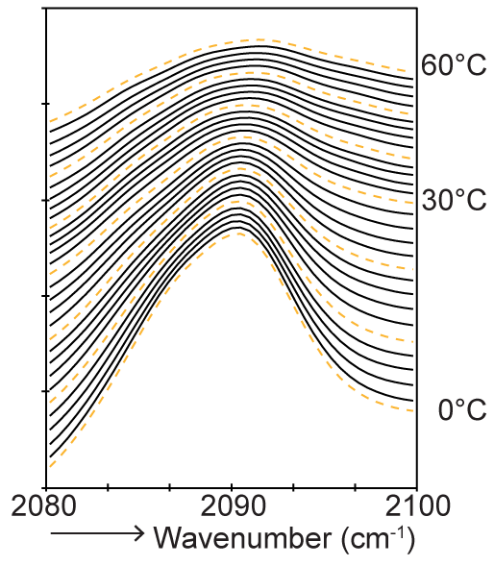


**Supplementary Figure 3.** FTIR spectrum showing CD_2_ symmetric stretching vibrations of Form^COMBI(dFA)^. The shape of the peak indicatest the presence of two types of vibrations, 2090 cm^-1^ and 2086 cm^-1^.

**
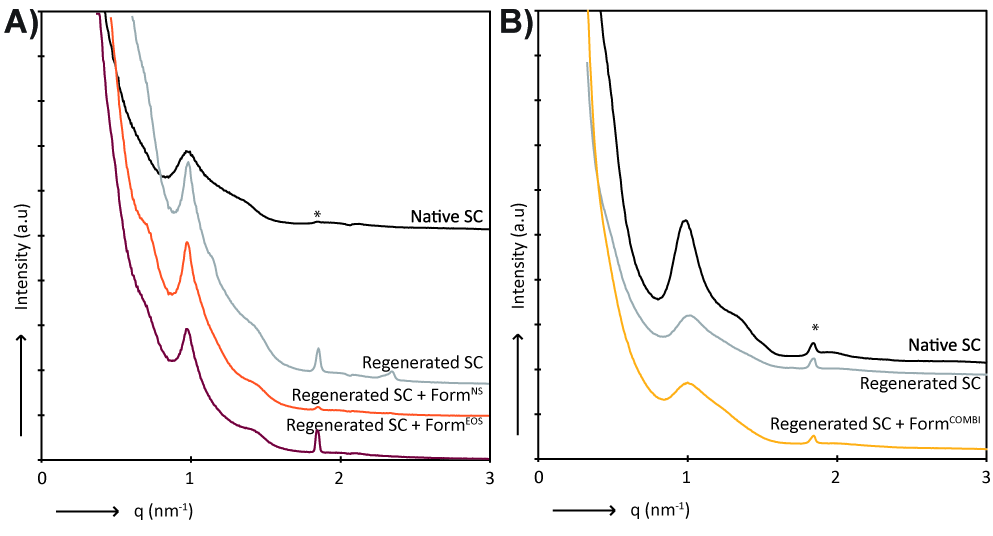
**

**Supplementary Figure 4.** X-ray diffraction patterns of native SC and regenerated SC with and without application of formulation. Both SPP and LPP attribute to the peaks at q=1. Phase separated CHOL is observed at q=1.8 and indicated by an asterisk. **A)** SAXD profiles of native SC, regenerated SC, and regenerated SC with application of Form^EOS^ and Form^NS^, **B)** SAXD profiles of native SC, regenerated SC, and regenerated SC with application of Form^COMBI^.

**References**

1. Danso MO, Berkers T, Mieremet A, Hausil F, Bouwstra JA. An ex vivo human skin model for studying skin barrier repair. Exp Dermatol. 2015;24(1):48-54.

2. Berkers T, van Dijk L, Absalah S, van Smeden J, Bouwstra JA. Topically applied fatty acids are elongated before incorporation in the stratum corneum lipid matrix in compromised skin. Exp Dermatol. 2016.

3. Damien F, Boncheva M. The extent of orthorhombic lipid phases in the stratum corneum determines the barrier efficiency of human skin in vivo. J Invest Dermatol. 2010;130(2):611-614.

4. Groen D, Gooris GS, Bouwstra JA. New insights into the stratum corneum lipid organization by X-ray diffraction analysis. Biophys J. 2009;97(8):2242-2249.
